# Integrating consumer engagement in health and medical research – an Australian framework

**DOI:** 10.1186/s12961-017-0171-2

**Published:** 2017-02-10

**Authors:** Caroline L. Miller, Kathy Mott, Michael Cousins, Stephanie Miller, Anne Johnson, Tony Lawson, Steve Wesselingh

**Affiliations:** 1 South Australian Health and Medical Research Institute (SAHMRI), North Terrace, Adelaide, South Australia 5000 Australia; 2 School of Public Health, University of Adelaide, Adelaide, Australia; 3 Samson Institute, University of South Australia, Adelaide, Australia; 4 Health Consumers Alliance of South Australia, Level 1, 12 Pirie Street, Adelaide, South Australia 5000 Australia; 5 320 Arthur Street, Penola, South Australia 5277 Australia

**Keywords:** Consumer engagement, Community participation, Community involvement

## Abstract

**Background:**

Quality practice of consumer engagement is still in its infancy in many sectors of medical research. The South Australian Health and Medical Research Institute (SAHMRI) identified, early in its development, the opportunity to integrate evidence-driven consumer and community engagement into its operations.

**Process:**

SAHMRI partnered with Health Consumers Alliance and consumers in evidence generation. A Partnership Steering Committee of researchers and consumers was formed for the project. An iterative mixed-method qualitative process was used to generate a framework for consumer engagement. This process included a literature review followed by semi-structured interviews with experts in consumer engagement and lead medical researchers, group discussions and a consensus workshop with the Partnership Steering Committee, facilitated by Health Consumer Alliance.

**Outcomes:**

The literature revealed a dearth of evidence about effective consumer engagement methodologies. Four organisational dimensions are reported to contribute to success, namely governance, infrastructure, capacity and advocacy. Key themes identified through the stakeholder interviews included sustained leadership, tangible benefits, engagement strategies should be varied, resourcing, a moral dimension, and challenges. The consensus workshop produced a framework and tangible strategies.

**Conclusion:**

Comprehensive examples of consumer participation in health and medical research are limited. There are few documented studies of what techniques are effective. This evidence-driven framework, developed in collaboration with consumers, is being integrated in a health and medical research institute with diverse programs of research. This framework is offered as a contribution to the evidence base around meaningful consumer engagement and as a template for other research institutions to utilise.

## Background

The active involvement of consumers and community in health, medical and biomedical research has become increasingly central to the research policy agenda of Australia, United Kingdom, Canada, United States of America, and elsewhere. Health and medical research is a matter of public interest, it is fundamentally designed to improve human health, and is predominantly funded through public resourcing, either via government research funds, or via charitable donations. Those resources are limited and there is a need to prioritise. Additionally, there is the core principle that those who are affected by research have a right to have a say in what is researched and how research is undertaken.

Despite wide acceptance of the potential benefits of involving consumers in health and medical research in Australia, there has been reluctance by some groups to test these potential benefits [1]. No implementation plan was developed to drive consumer participation in national research policy statements. This deficit, and the lack of structures and mechanisms to support consumer and community participation in health and medical research, were identified as factors that reduced the effectiveness of the policy direction and have led to ad hoc implementation [2]. Australia essentially lacks the range of structures and mechanisms found to be useful by other countries in supporting consumer and community participation into health and medical research [1]. A strategic review of health and medical research in Australia reported that consumer engagement in Australia requires leadership in order to achieve the outcome of consumers being meaningfully involved in initial stages of research, shaping research topics to improve the quality of research and research outcomes [3].

Continuation of the traditional health and medical research paradigm, in which research has been driven by researchers and ‘curiosity’, with the role of consumers as passive participants in research, has led to mismatches between the research that is undertaken and the research that consumers believe should be done, and renewed calls for greater involvement of consumers in the research processes. It has been argued that health and medical research is a social process, and as such it should be informed by the interactions of researchers and potential end beneficiaries, where both groups exchange expectations, views and ideas, and combine this knowledge to enhance the quality of the research [1].

There are three principal drivers for consumer and community participation in research:

- the political imperative for the engagement of consumers in research, due in large part to public and consumer interest in the research it funds, participates in and the outcomes of which it is affected by;
- the growth of consumer and carer advocacy, where the influence of consumer advocacy has been effective in ensuring that consumers are involved extensively in research;
- the academic community who involve consumers in research are promoting the benefits of such engagement [4, 5].

In addition to a political mandate, the pursuit of quality and relevant research is an important driver.

The South Australian Health and Medical Research Institute (SAHMRI) was a new institute, with an agenda of research excellence with a strong focus on research translation and a state-wide focus. South Australia has a state-wide Advanced Health Science and Translation Centre, led by SAHMRI.

SAHMRI identified, early in its development, the opportunity to integrate consumer and community engagement into its research and operations. The research within SAHMRI is diverse spanning seven different research themes (Heart Health, Cancer, Aboriginal Health, Child and Maternal Health, Infectious Diseases, Mental Health, and Nutrition and Metabolism) and four pillars (from bioscience to public health). It includes areas where consumer and community participation has been negligible through to those where participation has been a strong feature. The challenge was to integrate consumer engagement at the macro and organisational culture level, rather than at the micro or individual study level.

### Aim

SAHMRI undertook a process with the aim of developing an evidence-based, best-practice (consumer partnership), practical framework for consumer and community engagement in research process for the Institute, with a remit for an entire jurisdiction – the State of South Australia.

## Process

A mixed-method, iterative process was undertaken to identify available evidence, gather experiences and views of stakeholders (namely consumers, researchers, and academic experts in consumer engagement) and engage those stakeholders in the design of a consumer and community engagement framework for health and medical research. To further embed a consumer perspective in the development of the framework, a partnership was established with the local peak body for health consumers – Health Consumers Alliance of SA Inc. (HCA). A Partnership Steering Committee of scientists, researchers and consumers was formed for oversight of the project. Greater details can be accessed at: https://www.sahmri.org/consumer-community-engagement/.

### Literature review

The literature review was designed to answer the question, ‘What strategies for consumer engagement in health and medical research have been effective for consumers and researchers?’ An environmental scan of the grey and peer-reviewed literatures was undertaken. Search engines SciVerse Hub, Google and Google Scholar were utilised. Electronic biomedical databases searched were CINAHL, Pub-Med, Cochrane Library, OVID and ProQuest. Data were extracted from relevant links such as title, source, author, URL, content description and main conclusions. Years searched were 1988–2013 inclusive.

The search strategy and terms were developed with the input from the Project Steering Committee. Combinations of concepts were used as search terms for the structured literature review (Table 1).

**Table 1:** Search terms

Concept 1	Concept 2	Concept 3	Concept 4
Consumer	Participate	Health	Research
Community	Engage	Medical	Evaluation
Patient	Involve	Bio-medical	
Citizen	Consult	Animal	
Client	Empower		
User	Collaborate		
Lay	Inform		
Public			

Medical subject heading (MESH) terms and text words were selected based on the listed range of search concepts and adapted to common indexing practices for each database. Additional searches were conducted using the PubMed ‘related articles’ feature. Reference lists from germinal articles (systematic reviews and literature reviews) were reviewed and a search undertaken for relevant articles. In addition, Health Expectations (an international journal of public participation in healthcare and health policy) was hand searched because this journal appeared to have more articles on the topic than any other identified in the literature search through the databases.

A total of 268 references were identified and sourced. After assessing their relevance, 168 articles were included in the literature review.

### Consultation and development processes

In-depth, semi-structured interviews were conducted with key stakeholders (n = 22) identified by the Partnership Steering Committee. These included three informant groups, namely consumers and carers, academics and engagement practitioners with significant experience, and SAHMRI Research Theme Leaders. The purpose of the interviews was to gain insight from those stakeholders about the practice of consumer engagement to add to the literature review, but also to a framework for consumer engagement, using co-design, which would be relevant and acceptable to its intended audience. The interviews lasted approximately 45 minutes. They focussed on experience of effective consumer engagement, practical challenges experienced, and perceived opportunities and aspirations for optimal consumer and community engagement. The interviews were recorded and interviews were analysed for key themes by two authors and represented back to interview participants for validation.

Facilitated group discussions were held with the Partnership Steering Committee at the start of the project, after the literature review and after the stakeholder interviews. The process ended with a full day consensus workshop held with SAHMRI research leadership, consumers and researchers (n = 22; 13 researchers and 9 consumers). The workshop was facilitated by Health Consumers Alliance, presented the findings from the stages of the process to date, and was structured to yield a Framework which incorporated evidence gathered and included principles, elements and actions proposed and agreed by the Partnership Steering Committee.

## Outcomes

One of the criticisms in the academic literature was the lack of an evidence-based framework for consumer and community participation in health and medical research [6]. The literature review was designed to investigate which strategies for consumer engagement in health and medical research had been found to be effective. It was evident from the review that effectiveness of strategies used in consumer and community engagement in health and medical research is highly context-specific, and in many instances dependent on the attitudes, skill, and relationships between the consumers and researchers involved in the research process. Evaluation of strategies and comparative studies were hampered by lack of evaluation frameworks, as well as due to contextual issues such as policy and variations in utilisation of terminology, ideology, models of participation and methodology. There is variation in how the evidence of effectiveness of different strategies of consumer and community participation is evaluated and reported [6–8]. Many of the studies reviewed were qualitative in design and may not carry the same weight of evidence within the positivist paradigm of health and medical research [9]. There is generally inadequate reporting with a lack of valid and reliable tools [10]. Guidelines for the reporting of consumer and community engagement could improve consistency and comparability of studies.

The review identified two levels of organisational and research program activities that should be considered for consumer and community engagement in research (1) the conditions within research organisations that foster and support consumer and community engagement, and (2) strategies and actions used in a research program to enable consumer and community participation.

Four organisational dimensions are reported to contribute to success in consumer and community engagement, namely governance, infrastructure, capacity and advocacy [2].

### Governance

- Structures: concerted efforts through the establishment of shared supportive structures.
- Policy: comprehensive organisation-wide policy, including acknowledgement of consumers as key stakeholders in all research; partnership roles decided through consultation between consumers, community and researchers, which are based on mutual respect for one another’s different knowledge and experience; and resources including a practice guide to support policy implementation.
- Research funding: ensuring that consumers have an influential and sustained voice in research funding decisions.

### Infrastructure

- Consumer registers: registers of consumers, with experience working in research, advocacy and policy development, interested in research decision-making and support.
- Information: formal and informal support networks and resources and opportunity for consumers to share information and advice.

### Capacity

- Consumer training: adequate support through training, education and resources appropriate to the expected roles.
- Researcher training: program for researchers to better understand the contribution that the community can make to the research as active partners.

### Advocacy

- As a research organisation, actively promoting and advocating for greater consumer participation in health and medical research.

Key themes identified through the stakeholder interviews included the importance of sustained leadership, the benefits of increased consumer engagement, that consumer engagement has a moral dimension that suggests it should be mandatory, and that consumer engagement should be varied and appropriate – there is no one size that fits all needs.

Challenges that emerged were that successful consumer engagement requires resources and funding, there are barriers to participation, how should consumer engagement adequately reflect the diversity of society, there are entrenched attitudes (positive and negative) to consumer participation, and there is divergence between a social view of health and a biomedical view of health.

The consensus workshop was used to reach agreement on a proposed framework and general endorsement of the development of a broad range of strategies to integrate consumer and community engagement in the research conducted at SAHMRI, building upon the findings of the literature review, stakeholder interviews and Partnership Steering Committee discussions.

**Fig. 1:**
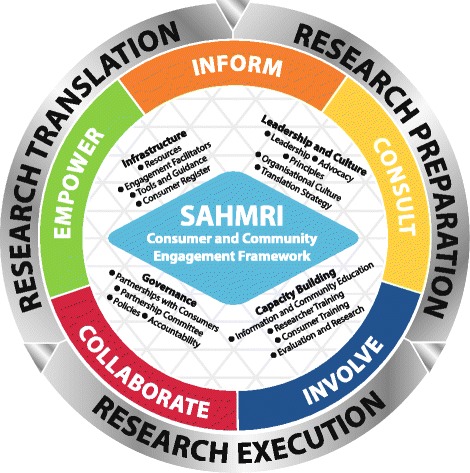
SAHMRI’s framework for consumer and community engagement in research

The resultant Consumer and Community Engagement Framework incorporates the four organisational domains – governance, infrastructure, capacity building, and leadership and culture [2], the International Association of Public Participation’s Levels of Participation, from the basic level of informing through to the ultimate level of empowering, and the phases of health and medical research [7]. A diagram summarising the Framework is provided in Fig. 1.

The Framework includes 17 operational elements, grouped under the four organisational domains. Five early ‘wins” were identified to kick-start the implementation of the Framework, and to build the culture of acceptance and expectation of consumer participation in Institute research. These are (1) a series of community conversations, research forums and research showcases; (2) further develop strategic partnerships with consumer and community organisations aligned to each of the seven SAHMRI Research Themes; (3) develop a series of research priority setting partnerships; (4) make consumer engagement mandatory in all grant applications; and (5) incorporate consumer and community engagement into the organisational statement on values and culture.

A set of Principles, considered essential to underpin Framework, were agreed. These are that consumer and community engagement in health and medical research:

- is based on the understanding that those affected by research have a right to be involved in all aspects of research, from being research participants in studies, through to research priority setting and research governance;
- can and should, where possible, occur across all phases and stages of research;
- is based on partnerships between consumers, the community and researchers to determine research priorities;
- includes the promise that consumer and community contributions will influence research;
- is sustainable by explicitly acknowledging the needs and interests of all stakeholders;
- actively facilitates involvement, practically supports participation and seeks input from research participants in designing how they participate;
- communicates to participants how their input influences research (based on rigorous evaluation);
- provides opportunities for consumers, communities and community organisations to develop their capacity, abilities and skills.

## Discussion

It is clear that, for consumer and community participation to be a reality, and to be integrated and effective in a research institution, each of the four organisational domains of governance, infrastructure, capacity and advocacy must be addressed [2]. Together, they deliver the environment, systems and culture that enable individuals (consumers, researchers, research leaders, administrators and managers) to implement the principles and Framework identified and agreed upon. A strong organisational will and commitment is necessary.

To date, there have been limited examples in Australia of a comprehensive systemic approach to consumer participation in health and medical research. There have been pockets of success, generally in areas where there is a groundswell of activism around a health condition (e.g. HIV, breast cancer) or a disadvantaged community (Aboriginal health) [11–13]. However, there has been limited effort and almost no documented studies of participation of consumers in the more traditional scientific research endeavours. There are also few documented examples where a whole Institute or whole of jurisdiction approach has been considered or implemented.

Challenges and opportunities exist to embed consumer participation across a very wide range of research pursuits and in a multitude of ways in a new research establishment such as SAHMRI. The enthusiasm and positive attitude shown by the cross-section of researchers involved in the consumer engagement project at SAHMRI has provided the basis for a bold experiment in research planning, implementation, governance and accountability. The opportunity to embed evaluation into the framework will be critical and assist with informing the evidence base.

The strengths of the Framework are its basis in evidence and its co-design process. The limitations of the Framework will be clearer in its implementation. For example, the recommendation to make consumer engagement a responsibility for all staff and incorporated into accountability processes within SAHMRI as a key performance indicator at Theme level will present significant challenges for the organisation and some Research Themes. As the Framework is implemented, the objective measurement, and the general narrative of how the Institute as a whole, individual researchers and their teams respond and perform will be critical to measure the Framework’s success and also building the evidence base for consumer participation in health and medical research. Qualitative studies with consumers will be embedded within the processes to evaluate engagement strategies and the meaningfulness of that engagement.

## Conclusion

The purpose of this process was to develop a workable Framework for consumer and community engagement for a new medical research institute with broader applicability. The Framework was designed using the best-available evidence and through a process of joint design between consumers and researchers, facilitated and enabled by a peak consumer agency. The Framework and Principles developed to underpin and guide SAHMRI’s consumer and community engagement approach and activities are innovative, comprehensive and practical. It is designed to articulate the reasons for and the ways to integrate consumer and community engagement in medical research from the individual research program to a whole of institute level. Its subsequent evaluation will build evidence about effectiveness in a field where that has been found to be wanting. It may be beneficial for other research organisations to consider these outcomes of the extensive process that SAHMRI has undertaken to gain clarity for their own framework and principles for consumer and community engagement.

## Abbreviations

HCA: Health Consumers Alliance of SA Inc.
SAHMRI: South Australian Health and Medical Research Institute
